# Short-Chain Fatty Acids in Blood Pressure Management: A Systematic Review of Clinical Effects, Delivery Strategies, and Translational Barriers

**DOI:** 10.7759/cureus.115011

**Published:** 2026-08-23

**Authors:** Nitin Wathore, Sharanya Kumbam, David Ibikunle, Mirna U Abdelrahman, Chinmay M Kalakonda, Ginika P Ejimgini, Rabia Azhar, Samiya Khanam, Allah Bux Ali

**Affiliations:** 1 Internal Medicine, Gulf Medical University, Ajman, ARE; 2 Internal Medicine, Windsor University School of Medicine, Cayon, KNA; 3 Internal Medicine, Redeemer's Health Centre, Ibadan, NGA; 4 Gastroenterology and Hepatology, Obafemi Awolowo University Teaching Hospitals Complex, Ile-Ife, NGA; 5 Internal Medicine, Alexandria University, Alexandria, EGY; 6 Biological Sciences, University of Massachusetts Amherst, Amherst, USA; 7 Internal Medicine, Nnamdi Azikiwe University, Awka, NGA; 8 Internal Medicine, Lahore Medical and Dental College, Lahore, PAK; 9 Internal Medicine, Batterjee Medical College, Jeddah, SAU; 10 Internal Medicine, Sir Ganga Ram Hospital, Lahore, PAK

**Keywords:** acetate, ambulatory blood pressure monitoring, blood pressure, butyrate, gut microbiome, hypertension, propionate, short-chain fatty acids

## Abstract

Short-chain fatty acids (SCFAs) have been proposed as mediators of the relationship between the intestinal microbiome and blood pressure (BP), but their therapeutic relevance in humans remains uncertain. This systematic review evaluated the effects of direct SCFA administration and interventions modifying endogenous fecal or circulating SCFAs on BP in adults. MEDLINE via PubMed, Scopus, and Web of Science Core Collection were searched for randomized controlled trials published from January 1, 2022, through May 31, 2026. The review was reported in accordance with the Preferred Reporting Items for Systematic Reviews and Meta-Analyses (PRISMA) 2020 statement, with study selection, data extraction, and risk-of-bias assessment performed independently by two reviewers. Six randomized controlled trials were included and synthesized narratively because of substantial clinical and methodological heterogeneity. Three trials evaluated direct butyrate administration, while three assessed exercise, plant-derived, dietary, prebiotic, or physical therapy interventions that modified endogenous SCFAs. Direct administration produced inconsistent effects. Conventional oral butyrate increased daytime systolic and diastolic BP relative to placebo in adults with hypertension, whereas acute colon-targeted delivery reduced daytime systolic BP relative to a lower concentration in a small crossover trial. A further oral trial in type 2 diabetes mellitus reported within-group BP reductions without a demonstrated between-group effect. Indirect interventions generally increased plasma or fecal SCFAs alongside improvements in selected BP outcomes, but their multicomponent effects and the absence of formal mediation analyses precluded attributing these responses specifically to SCFAs. Fecal and circulating measurements also showed different relationships with BP, indicating that these compartments are not biologically interchangeable. One study was judged at low risk of bias, two had some concerns, and three were at high risk. Current evidence does not demonstrate a uniform antihypertensive effect of increasing SCFA availability or a reproducible dose-response relationship. Future trials should compare oral and colon-targeted formulations, measure fecal and circulating SCFAs concurrently, use 24-hour ambulatory BP monitoring as the primary outcome, and evaluate whether treatment responses vary by exposure and cardiometabolic phenotype.

## Introduction and background

Hypertension remains a major preventable contributor to cardiovascular, cerebrovascular, and renal morbidity, yet adequate blood pressure control is not consistently achieved despite the availability of effective pharmacological and lifestyle interventions. This continuing burden has encouraged investigation of biological pathways that may complement established approaches to treatment [1]. The intestinal microbiome has emerged as a potential contributor to blood pressure regulation through its interactions with dietary substrates, immune signaling, vascular function, and host metabolism. Among the metabolites generated by intestinal microorganisms, short-chain fatty acids (SCFAs) have attracted particular interest because they provide a plausible biochemical link between dietary fiber, microbial activity, and cardiovascular physiology. However, the clinical relevance of this pathway remains uncertain, and associations between microbial composition, SCFA concentrations, and blood pressure do not independently establish a modifiable causal relationship [2].

Acetate, propionate, and butyrate are the principal SCFAs produced through microbial fermentation of otherwise indigestible carbohydrates. After production in the colon, these metabolites may be utilized locally, absorbed into the portal circulation, metabolized by the liver, or distributed to peripheral tissues. SCFAs may influence vascular tone, endothelial nitric oxide signaling, renin release, renal sodium handling, sympathetic activity, inflammation, and cellular metabolism through several receptor-dependent and receptor-independent pathways. Importantly, these effects may not operate uniformly [3,4]. Differences in receptor activation and tissue distribution may generate opposing vascular or renal responses, while the net physiological effect may vary according to concentration, route of exposure, and cardiometabolic state. Interpretation is further complicated by the biological distinction between fecal and circulating SCFAs [5]. Fecal concentrations represent the residual balance among production, absorption, microbial utilization, colonic transit, and excretion, whereas circulating concentrations reflect absorbed exposure after intestinal and hepatic metabolism. Neither compartment can therefore be regarded as a complete measure of SCFA production or biological activity [6].

Preclinical studies have frequently reported favorable effects of SCFA supplementation or enhanced microbial SCFA production on blood pressure, endothelial function, and inflammation. Translation into human hypertension management, however, has been inconsistent. Direct supplementation may produce different effects depending on whether an SCFA is administered orally or delivered directly to the colon, while the administered dose may not translate proportionally into local intestinal or systemic exposure [7]. Indirect strategies, including exercise, dietary fiber, prebiotics, and plant-derived interventions, may increase fecal or circulating SCFAs alongside improvements in blood pressure. However, these interventions also influence numerous cardiovascular pathways independently of SCFAs, making causal mediation difficult to establish. Differences in hypertension status, type 2 diabetes mellitus, concomitant medication, intervention duration, SCFA measurement, and blood pressure assessment further limit comparisons across studies. A synthesis that treats all SCFA-related interventions as biologically equivalent may therefore obscure clinically important distinctions in delivery route, biological compartment, and host phenotype.

This systematic review aimed to evaluate the effects of direct SCFA administration and interventions that modify endogenous fecal or circulating SCFAs on blood pressure in adults. It compared the direction and magnitude of blood pressure responses across intervention strategies, with particular attention to SCFA species, dose, delivery route, biological compartment, blood pressure measurement, and cardiometabolic phenotype. By separating direct-administration trials from indirect SCFA-modifying interventions, the review sought to distinguish evidence of treatment efficacy from evidence of concurrent metabolic change, assess the certainty and methodological limitations of the available randomized evidence, and identify the principal barriers that must be addressed before SCFA-targeted approaches can be translated into safe and reproducible hypertension therapy.

## Review

Materials and methods

Review Design and Reporting Framework

This systematic review evaluated the effects of direct SCFA administration and interventions that modify endogenous SCFA availability on blood pressure in adults. The review was conducted and reported in accordance with the Preferred Reporting Items for Systematic Reviews and Meta-Analyses (PRISMA) 2020 statement [8]. Before formal screening, a review protocol was developed specifying the research question, eligibility criteria, search strategy, study-selection process, outcomes, risk-of-bias assessment, and synthesis plan. The numbers of records identified, removed as duplicates, screened, assessed in full text, and excluded with reasons are presented in the PRISMA 2020 flow diagram.

Research Question and PICO Framework

The review question was formulated using the Population, Intervention, Comparator, and Outcome (PICO) framework [9]. The population comprised adults aged 18 years or older with hypertension or a cardiometabolic phenotype associated with increased hypertension risk, including type 2 diabetes mellitus. Eligible interventions included direct administration of acetate, propionate, butyrate, or another SCFA by any route, as well as dietary, prebiotic, exercise, plant-derived, or physical therapy interventions that measured corresponding changes in fecal or circulating SCFAs. Comparators included placebo, a lower SCFA concentration, usual care, no intervention, standard antihypertensive therapy, or an active pharmacological comparator. The principal outcome was change in systolic or diastolic blood pressure measured using validated office or ambulatory methods. Secondary outcomes included changes in fecal or circulating SCFAs, associations between SCFA and blood pressure changes, arterial stiffness, adverse events, and intervention tolerability.

Information Sources and Search Period

MEDLINE via PubMed, Scopus, and Web of Science Core Collection were systematically searched for studies published from January 1, 2022, through May 31, 2026. The search combined controlled vocabulary, including Medical Subject Headings where applicable, with free-text terms. SCFA-related terms included “short-chain fatty acids,” “SCFAs,” “volatile fatty acids,” “acetate,” “acetic acid,” “propionate,” “propionic acid,” “butyrate,” “butyric acid,” and “sodium butyrate.” Blood pressure terms included “hypertension,” “high blood pressure,” “blood pressure,” “systolic,” “diastolic,” and “ambulatory blood pressure.” Study-design terms included “randomized controlled trial,” “randomized,” “randomised,” “placebo,” “crossover,” “cross-over,” and “intervention.” The Boolean operator OR was used to combine synonymous terms within each concept, while AND was used to intersect the SCFA, blood pressure, and randomized-intervention concepts. Truncation and phrase searching were applied where supported to capture alternative word endings and exact multiword terms. Search syntax was adapted to the indexing conventions of each database while preserving the same conceptual structure. Reference lists of included studies and relevant reviews were examined for additional eligible reports. No language restrictions were applied.

Eligibility Criteria

Randomized controlled trials using parallel-group, crossover, or multi-arm designs were eligible if they enrolled adults aged 18 years or older with hypertension or a relevant cardiometabolic phenotype, including type 2 diabetes mellitus, and reported a quantitative systolic or diastolic blood pressure outcome. Direct-intervention trials were eligible when acetate, propionate, butyrate, or another SCFA was administered through a defined route, regardless of whether post-intervention SCFA concentrations were measured. Indirect-intervention trials involving exercise, dietary, prebiotic, plant-derived, or physical therapy strategies were eligible only when fecal or circulating acetate, propionate, butyrate, or total SCFAs were measured before and after the intervention. This distinction permitted evaluation of direct SCFA efficacy while preventing the inclusion of general lifestyle or metabolic trials without documented SCFA assessment.

Eligible comparators included placebo, sodium-matched placebo, a lower SCFA concentration, usual care, no intervention, standard antihypertensive therapy, or an active pharmacological comparator. Blood pressure could be a primary or secondary outcome and could be assessed using office measurement or ambulatory monitoring. Animal and in vitro studies, observational studies, nonrandomized interventions, case reports, case series, reviews, editorials, protocols, and conference abstracts without sufficient outcome data were excluded. Studies reporting microbiome composition without direct SCFA administration or quantitative SCFA measurement were excluded, as were studies measuring SCFAs without reporting systolic or diastolic blood pressure. Compounds containing “acetate” in their chemical names but unrelated to microbiota-derived acetate, such as desmopressin acetate and abiraterone acetate, were also excluded.

Study Selection

All retrieved records were combined, and duplicate entries were removed before screening. Two reviewers independently screened titles and abstracts against the predefined eligibility criteria. Records considered potentially relevant by either reviewer proceeded to full-text assessment. The same reviewers independently evaluated the complete reports and documented the principal reason for excluding each ineligible article. Disagreements were resolved through discussion and consensus, with consultation of a third reviewer when consensus could not be reached. Multiple publications arising from the same trial were linked and treated as a single study, with the report containing the most complete blood pressure and SCFA data designated as the primary source.

Data Extraction

Two reviewers independently extracted data using a standardized, pilot-tested form. Extracted information included author, publication year, country, study design, sample size, participant age and sex, hypertension or metabolic phenotype, baseline blood pressure, antihypertensive medication status, intervention composition, SCFA species, dose, route, duration, comparator and follow-up period. SCFA-related data included the measured biological compartment, analytical method, sampling time and changes in acetate, propionate, butyrate and total SCFAs. Blood pressure data included the measurement technique, monitoring period, primary or secondary outcome status, baseline and follow-up values, within-group changes, between-group differences, confidence intervals and P values. Information on adherence, withdrawals, adverse events, funding and trial registration was also collected. Disagreements were resolved by consensus or adjudication by a third reviewer.

Outcomes and Effect Estimates

The principal outcomes were between-group differences in systolic and diastolic blood pressure following intervention. Twenty-four-hour ambulatory measurements were prioritized over office measurements when both were available, followed by prespecified daytime and nighttime outcomes. Between-group estimates were prioritized because within-group statistical significance does not establish a randomized treatment effect. When a study reported only within-group results, these findings were extracted but explicitly identified as uncontrolled pre-post changes. For SCFA outcomes, changes in individual and total SCFA concentrations were recorded separately according to whether they were measured in plasma, serum, whole blood or feces. Reported associations between changes in SCFAs and blood pressure were extracted without interpreting correlation as evidence of causal mediation.

Risk of Bias Assessment

Risk of bias was evaluated independently by two reviewers using the Cochrane Risk of Bias 2 (RoB 2) tool for randomized trials [10]. The standard version was applied to parallel-group and multi-arm trials, while the crossover version was applied to crossover studies. Assessment was performed specifically for the blood pressure result and covered the randomization process, deviations from intended interventions, missing outcome data, measurement of the outcome, and selection of the reported result. Each domain and the overall result were classified as low risk, some concerns, or high risk of bias. Disagreements were resolved by consensus, with adjudication by a third reviewer when required. Small sample size was considered a limitation of precision but was not itself used to assign a higher risk-of-bias judgment.

Data Synthesis

Studies were grouped into direct SCFA-administration trials and interventions modifying endogenous fecal or circulating SCFAs. A structured narrative synthesis compared populations, delivery routes, SCFA compartments, treatment duration, blood pressure methods and risk of bias, with emphasis on between-group effects. Meta-analysis was not performed because substantial clinical and methodological heterogeneity precluded estimation of a common treatment effect. SCFA changes accompanying blood pressure responses were not interpreted as causal without formal mediation analysis.

Ethical Considerations

Because this review used data from previously published studies and involved no direct participation of human subjects, institutional ethical approval and individual informed consent were not required.

Results

Study Selection Process

**Figure 1:**
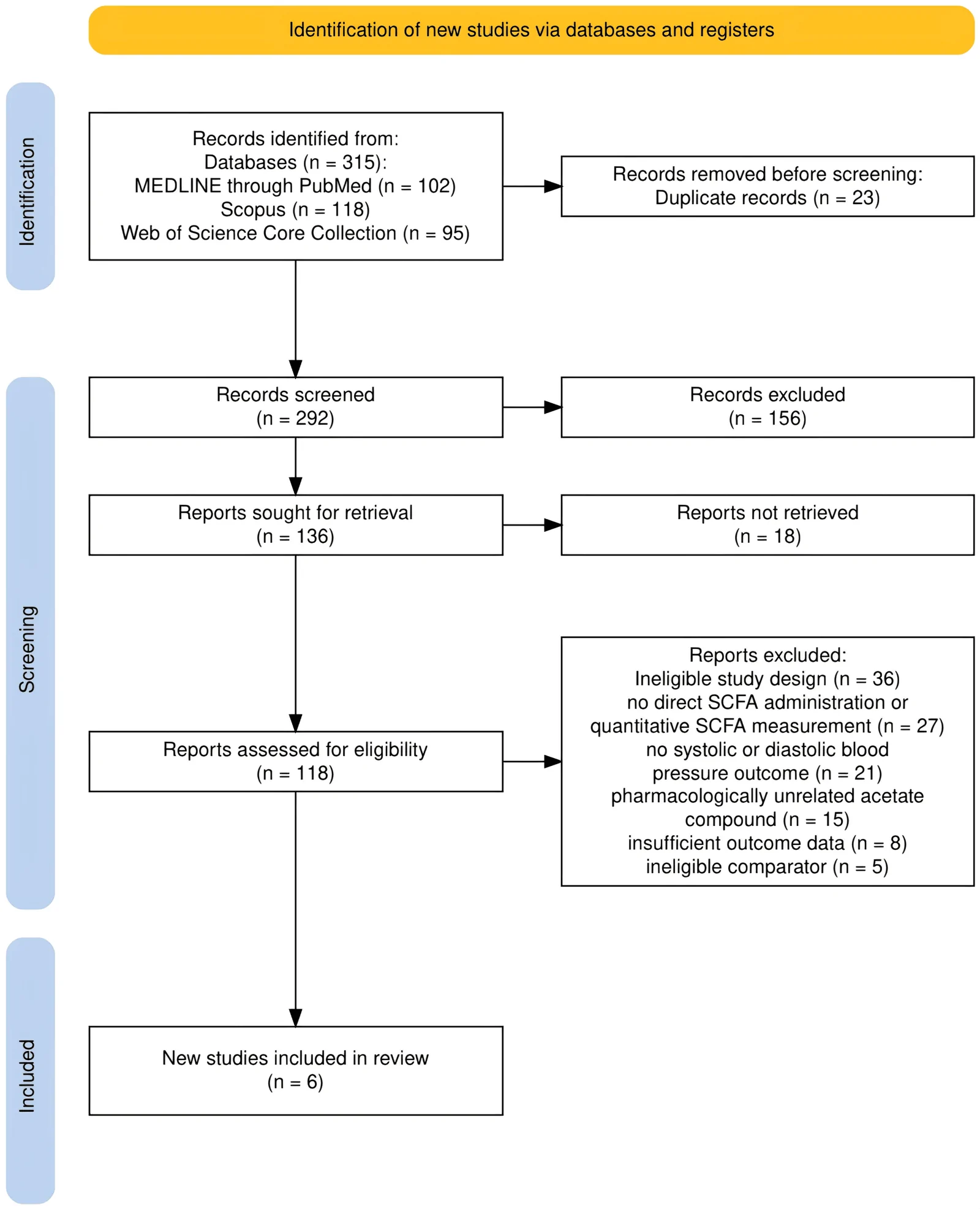
PRISMA 2020 flow diagram of study identification, screening, eligibility assessment, and inclusion. PRISMA: Preferred Reporting Items for Systematic Reviews and Meta-Analyses [8]; SCFA: short-chain fatty acid

The database search identified 315 records, of which 23 duplicates were removed, leaving 292 records for title and abstract screening. After excluding 156 records, 136 reports were sought for retrieval, of which 18 could not be obtained. The remaining 118 full-text reports were assessed for eligibility, and 112 were excluded for predefined reasons. Ultimately, six randomized studies met the eligibility criteria and were included in the systematic review (Figure 1).

Study Characteristics

Six randomized studies involving 267 participants were included (Table 1). Three evaluated direct butyrate administration through oral supplementation or colon-targeted delivery, while three examined exercise, plant-derived, prebiotic, dietary or physical-therapy interventions that modified endogenous SCFAs. Populations included adults with hypertension and type 2 diabetes, with intervention durations ranging from a single acute exposure to 12 months. Blood pressure was assessed using office measurement, 24-hour ambulatory monitoring or both, while SCFAs were measured in plasma, feces or not measured following direct supplementation.

**Table 1:** Summary of randomized controlled trials evaluating short-chain fatty acid-based interventions for blood pressure management in adults. SCFA: short-chain fatty acid; RCT: randomized controlled trial; BP: blood pressure; SBP: systolic blood pressure; DBP: diastolic blood pressure; ABPM: ambulatory blood pressure monitoring; HIIT: high-intensity interval training; T2DM: type 2 diabetes mellitus; CP: Cyclocarya paliurus; cfPWV: carotid-femoral pulse wave velocity; GC-MS: gas chromatography-mass spectrometry; CAVI: cardio-ankle vascular index; NR: not reported; CI: confidence interval

Study	Design and population	Intervention and comparator	SCFA strategy	SCFA measurement	BP assessment	Principal SCFA findings	Principal BP findings
Verhaar et al., 2024 [11]	Double-blind parallel RCT; 23 adults with hypertension randomized, 21 analyzed; mean age 59.0 years; antihypertensives withdrawn	Oral sodium butyrate 3.9 g/day vs sodium-matched placebo for 4 weeks	Direct oral butyrate	Plasma and fecal SCFAs at baseline and week 4	Office BP and 24-hour ABPM; primary outcome: daytime SBP	Plasma butyrate increased approximately 1.2-fold (P=.047); fecal butyrate did not significantly change	Butyrate increased daytime SBP by 9.63 mmHg (95% CI 2.02-17.20) and DBP by 5.08 mmHg (95% CI 1.34-8.78) versus placebo
Hogue et al., 2025 [12]	Proof-of-concept randomized crossover study; 10 Black adults with stage 1 hypertension received intervention; 10 matched normotensive adults were reference controls	Single high-concentration butyrate enema, 80 mmol/L, vs low concentration, 5 mmol/L; 7-day washout	Direct colon-targeted butyrate	Blood butyrate immediately before and 30 minutes after treatment; baseline fecal microbiome, but no fecal SCFA measurement	24-hour ABPM	Circulating butyrate did not change significantly after either concentration	High-concentration butyrate lowered daytime SBP by 4.6 mmHg versus low concentration (P=.034); 24-hour SBP difference was -4.0 mmHg (P=.054)
Ghardashi Afousi et al., 2026 [13]	12-week parallel RCT; 60 adults with hypertension; mean age 60.1 years	Cycling HIIT three times/week vs control	Indirect exercise-mediated SCFA modification	Plasma acetate, propionate and butyrate before and after treatment	Office BP and 24-hour ABPM; cfPWV also measured	HIIT increased acetate by 5.72±2.27, propionate by 0.76±0.36 and butyrate by 0.65±0.21 µmol/L (all P=.001); SCFA changes were associated with BP changes	Compared with control, HIIT reduced office BP by 6.9/3.1 mmHg and 24-hour BP by 6.1/3.5 mmHg (all P<.001)
Khosravi et al., 2022 [14]	Triple-blind parallel RCT; 42 adults with T2DM, 21 per group; hypertension status not specified	Oral sodium butyrate vs placebo for 6 weeks; dose NR in abstract	Direct oral butyrate	SCFA concentrations were not measured	BP before and after intervention; measurement method NR	No biological or pharmacokinetic confirmation of increased butyrate exposure	SBP and DBP decreased from baseline within the butyrate group (P=.016 and P=.002); no significant between-group BP effect was reported
Peng et al., 2023 [15]	Open-label pilot RCT; 38 treatment-naïve Chinese adults with newly diagnosed T2DM; CP n=25, glipizide n=13	Cyclocarya paliurus extract 2 g three times/day vs glipizide 5 mg twice/day for 84 days	Indirect microbiome-targeted SCFA modification	Fecal acetate, propionate, butyrate and total SCFAs at baseline and day 84	Office SBP and DBP; secondary outcomes	Total fecal SCFAs, propionate and butyrate increased within the CP group; acetate did not change; no direct SCFA-BP analysis	CP reduced SBP from 136.08 to 129.80 mmHg (P=.011) and median DBP from 81 to 74 mmHg (P=.001). Between-group change favored CP for DBP (P=.028), but not SBP (P=.820)
Petelina et al., 2025 [16]	Four-arm, 12-month RCT; 84 adults with grade 1-3 hypertension; mean group ages 47.7-53.5 years	Antihypertensive therapy alone or with high-fiber diet, fiber plus Plantago ovata, or fiber plus physical therapy	Indirect dietary, prebiotic and physical-therapy SCFA modification	Fecal SCFAs using GC-MS/GC with plasma-ionization detection	24-hour ABPM; PWV and CAVI also measured	Fecal propionate and butyrate increased in the prebiotic and physical-therapy arms; acetate findings NR	Physical-therapy arm showed reductions in 24-hour SBP (P=.043) and DBP (P=.028); effect sizes and between-group BP comparisons were not reported

Risk of Bias Assessment

One study was judged to have a low overall risk of bias, two raised some concerns and three were assessed as having a high risk of bias (Table 2). The principal concerns involved insufficient reporting of allocation procedures, unblinded or multicomponent interventions, incomplete information on missing data and selective presentation of blood pressure findings. Several studies emphasized within-group changes without adequate between-group estimates, while objective ambulatory blood pressure measurement reduced concern regarding outcome assessment in the relevant trials.

**Table 2:** Risk of bias assessment of randomized controlled trials evaluating short-chain fatty acid-based interventions for blood pressure management. RoB 2: Cochrane Risk of Bias 2 tool [10]; RCT: randomized controlled trial; BP: blood pressure

Study	RoB tool	D1: Randomization	D2: Deviations	D3: Missing data	D4: BP measurement	D5: Selective reporting	Overall
Verhaar et al., 2024 [11]	RoB 2 (parallel RCT)	Low	Low	Low	Low	Low	Low
Hogue et al., 2025 [12]	RoB 2 (crossover RCT)	Some concerns	Some concerns	Low	Low	Some concerns	Some concerns
Ghardashi Afousi et al., 2026 [13]	RoB 2 (parallel RCT)	Some concerns	Some concerns	Some concerns	Low	Some concerns	Some concerns
Khosravi et al., 2022 [14]	RoB 2 (parallel RCT)	Some concerns	Low	Some concerns	Some concerns	High	High
Peng et al., 2023 [15]	RoB 2 (parallel RCT)	Some concerns	Some concerns	Low	Some concerns	Some concerns	High
Petelina et al., 2025 [16]	RoB 2 (parallel four-arm RCT)	Some concerns	Some concerns	Some concerns	Low	High	High

Discussion

Principal Findings

This systematic review identified six randomized studies evaluating direct SCFA administration or interventions that modified endogenous SCFA concentrations in relation to blood pressure. Direct administration produced inconsistent findings. Conventional oral butyrate increased daytime systolic and diastolic blood pressure in one placebo-controlled trial involving adults with hypertension, whereas acute colon-targeted delivery reduced daytime systolic blood pressure in a small crossover study. A further oral supplementation trial reported reductions from baseline but did not demonstrate a clear treatment effect relative to placebo. Indirect interventions, including exercise, plant extract supplementation, dietary fiber, prebiotic therapy, and physical therapy, generally increased circulating or fecal SCFAs alongside improvements in selected blood pressure outcomes. However, differences in populations, delivery routes, intervention durations, comparators, SCFA compartments, and blood pressure assessment methods precluded estimation of a unified treatment effect. Collectively, the evidence does not support a uniform antihypertensive effect of increasing SCFA exposure. Rather, the observed response appears to depend on how and where SCFA availability is modified, as well as the intervention context and cardiometabolic phenotype of the treated population.

Direct Administration and the Delivery-Site Hypothesis

Among the direct-administration studies, Verhaar et al. [11] provided the most methodologically robust evidence through a double-blind, placebo-controlled design, withdrawal of antihypertensive medication, and 24-hour ambulatory blood pressure monitoring. Oral sodium butyrate increased plasma but not fecal butyrate and resulted in higher daytime systolic and diastolic blood pressure relative to placebo. By contrast, Hogue et al. [12] found that high-concentration butyrate delivered directly to the colon reduced daytime systolic blood pressure compared with a lower concentration, despite no corresponding increase in circulating butyrate. The trial by Khosravi et al. [14] reported significant within-group reductions in systolic and diastolic blood pressure following oral supplementation, but a significant between-group effect was not reported, limiting causal interpretation. Although these findings arose from small and clinically distinct samples, their divergence raises a plausible delivery-site hypothesis. Predominant absorption of orally administered butyrate before it reaches the colon may not reproduce the biological effects of local colonic exposure. The anatomical location at which butyrate becomes available may therefore be as important as the administered dose, although direct comparative trials are required to test this possibility.

The SCFA Compartment Problem

Interpretation is further complicated by the fact that fecal and circulating SCFAs represent related but biologically distinct compartments. Fecal concentrations reflect the residual balance among microbial production, intestinal absorption, microbial utilization, colonic transit, and excretion rather than production alone. Plasma concentrations more closely reflect absorbed systemic exposure but are influenced by intestinal uptake, hepatic first-pass metabolism, peripheral utilization, fasting status, and sampling time. This distinction was illustrated by Verhaar et al. [11], who observed an increase in plasma but not fecal butyrate, and by Hogue et al. [12], who detected a blood pressure response without a measurable elevation in circulating butyrate. The indirect studies also assessed different compartments. Ghardashi Afousi et al. [13] measured plasma acetate, propionate, and butyrate, whereas Peng et al. [15] and Petelina et al. [16] evaluated fecal SCFAs. Consequently, some apparent inconsistency across studies may reflect differences in the biological compartments assessed rather than genuinely opposing physiological effects. Future trials should measure fecal and circulating SCFAs concurrently using standardized sampling and analytical procedures rather than treating either compartment as a complete surrogate for SCFA production, absorption, or biological activity.

Indirect Interventions and the Problem of Causal Mediation

The indirect-intervention studies provide a distinct and more mechanistically complex evidence stream. Ghardashi Afousi et al. [13] found that high-intensity interval training increased plasma acetate, propionate and butyrate alongside reductions in office and 24-hour ambulatory blood pressure. Peng et al. [15] similarly reported that Cyclocarya paliurus supplementation increased fecal propionate, butyrate and total SCFAs while improving selected blood pressure outcomes. In the study by Petelina et al. [16], both prebiotic and physical-therapy interventions increased fecal propionate and butyrate, although reductions in mean 24-hour systolic and diastolic blood pressure were most evident in the physical-therapy group. These parallel changes are compatible with a potential role for SCFAs but are not sufficient to demonstrate mediation. Exercise, plant-derived compounds, dietary fiber, prebiotics and physical therapy may influence blood pressure through endothelial, autonomic, metabolic, inflammatory and vascular pathways that are partly or entirely independent of SCFAs. Importantly, randomization supports causal inference for the assigned intervention, not for a metabolite measured after randomization. These studies therefore position SCFAs as potential contributors to, or biomarkers of, a broader physiological response, but they do not establish that increased SCFA availability caused the observed blood pressure reductions.

Dose-Response and Phenotype Dependence

A clinically interpretable dose-response relationship cannot yet be defined from the available evidence. Hogue et al. [12] provided the most direct concentration comparison, with greater daytime systolic blood pressure reduction following an 80 mmol/L rather than a 5 mmol/L butyrate enema. However, only two acute concentrations were evaluated in 10 participants with hypertension, the lower concentration was biologically active rather than an inert placebo, and circulating butyrate did not demonstrate a corresponding exposure gradient. Comparisons across studies are even less informative because oral capsules, colonic enemas, exercise and microbiome-targeted interventions differ substantially in absorption, tissue distribution, metabolism and duration of exposure. Clinical heterogeneity adds further uncertainty. The evidence included adults with established hypertension, Black adults with stage 1 hypertension and treatment-naïve patients with type 2 diabetes mellitus, with additional variation in baseline blood pressure and cardiometabolic status. Antihypertensive medication was withdrawn in Verhaar et al. [11], continued as background therapy in Petelina et al. [16], and was not central to the diabetic trials. These differences could plausibly modify treatment responses. The evidence therefore demonstrates variation in administered dose and participant phenotype, but it does not establish a reproducible dose-response relationship or identify a reliable responder phenotype.

Biological Interpretation of the Divergent Findings

The divergent clinical findings are biologically plausible because SCFAs participate in several signaling and metabolic pathways rather than acting through a single blood pressure mechanism. Acetate, propionate and butyrate can interact with G protein-coupled receptors, including FFAR2, FFAR3 and olfactory receptor 78, and may also influence cellular function through histone deacetylase inhibition and metabolic substrate activity [17]. Experimental evidence suggests potential effects on vascular relaxation, endothelial nitric oxide signaling, renin release, renal sodium handling, sympathetic activity and immune regulation. These pathways need not operate in the same direction. Activation of selected free fatty acid receptors may promote vasodilation, whereas signaling through olfactory receptor 78 has been associated with renin release and a counter-regulatory increase in blood pressure in preclinical models. The net response could therefore vary according to SCFA species, local concentration, tissue distribution, receptor expression and underlying renal, vascular or metabolic state. Much of this mechanistic evidence remains derived from experimental models, so direct extrapolation to humans requires caution [18]. Nevertheless, it offers a coherent framework for understanding why increased SCFA availability may accompany either higher or lower blood pressure. SCFAs may consequently be better regarded as context-dependent signaling molecules than as uniformly protective cardiovascular metabolites.

Evidence Certainty and Review Limitations

The conclusions of this review must be interpreted within the limited certainty of the available evidence. Only one study was judged to have a low overall risk of bias, two raised some concerns and three were considered at high risk, principally because of incomplete methodological reporting, open-label or multicomponent interventions and selective or inadequate presentation of blood pressure results. Sample sizes were small, and the studies differed in population, intervention, comparator, duration and SCFA compartment. Blood pressure was a secondary outcome in several trials, while some reports emphasized significant within-group changes without presenting adequate between-group treatment estimates. SCFA measurement methods, biospecimen selection and sampling schedules were also inconsistent, limiting direct comparisons of biological exposure. None of the indirect studies performed a formal mediation analysis capable of quantifying whether SCFA changes accounted for any portion of the blood pressure response. A meta-analysis was therefore considered inappropriate because the studies did not estimate a sufficiently common intervention effect. At the review level, the small evidence base limited assessment of publication bias, while incomplete reporting and language-related accessibility may have constrained detailed evaluation of some trials. These limitations do not invalidate the observed findings, but they substantially restrict their precision, causal interpretation and generalizability.

Translational Implications and Future Research

The principal translational implication of this review is that SCFA-centered hypertension research should move beyond testing whether greater overall SCFA exposure lowers blood pressure and instead define the conditions under which a clinically meaningful response occurs. Future randomized trials should directly compare conventional oral and colon-targeted formulations across multiple doses, while measuring fecal and plasma acetate, propionate, and butyrate concurrently [19]. Repeated sampling will be necessary to characterize absorption, systemic exposure and temporal relationships with blood pressure. Twenty-four-hour ambulatory blood pressure should serve as the primary outcome, with daytime and nighttime results prespecified, and diet, sodium intake and antihypertensive therapy should be standardized to reduce competing influences. Trials should also prespecify subgroup analyses according to hypertension phenotype, metabolic status, ethnicity and baseline SCFA profile, while formal mediation analyses should determine whether SCFA changes account for any observed treatment effect. Longer follow-up is required to establish durability, tolerability and safety. SCFAs remain biologically credible targets in blood pressure regulation, but current evidence is insufficient to support their incorporation into routine hypertension management. The central translational question is therefore not simply whether SCFAs lower blood pressure, but which SCFA, delivered to which biological compartment, at what exposure and in which patient phenotype can produce a safe, sustained and reproducible response [20].

## Conclusions

Current human evidence does not support a uniform antihypertensive effect of increasing SCFA availability. Direct butyrate administration produced divergent blood pressure responses, whereas indirect interventions generally increased SCFAs alongside improved blood pressure without establishing SCFAs as causal mediators. These findings suggest that delivery route, anatomical site of exposure, biological compartment, and cardiometabolic phenotype may influence the direction and magnitude of response. The absence of a reproducible dose-response relationship, together with methodological heterogeneity and limited certainty, precludes the routine clinical use of SCFA-targeted therapy for hypertension. Nevertheless, the observed differences between conventional oral and colon-targeted delivery highlight an important translational priority. The principal take-home message is that SCFAs should not be regarded as uniformly beneficial metabolites. Their clinical value will depend on identifying the appropriate compound, delivery site, exposure, and patient phenotype required to produce a safe, sustained, and reproducible blood pressure response.
